# Accelerating the Classification of NOVA Food Processing Levels Using a Fine-Tuned Language Model: A Multi-Country Study

**DOI:** 10.3390/nu15194167

**Published:** 2023-09-27

**Authors:** Guanlan Hu, Nadia Flexner, María Victoria Tiscornia, Mary R. L’Abbé

**Affiliations:** 1 Department of Nutritional Sciences, Temerty Faculty of Medicine, University of Toronto, Toronto, ON M5S 1A1, Canada; guanlan.hu@utoronto.ca (G.H.); nadia.flexner@mail.utoronto.ca (N.F.); 2 Fundación Interamericana del Corazón Argentina, Buenos Aires C1425, Argentina; victoria.tiscornia@ficargentina.org

**Keywords:** ultra-processed foods, natural language processing, machine learning, food label, food composition database, NOVA system

## Abstract

The consumption and availability of ultra-processed foods (UPFs), which are associated with an increased risk of noncommunicable diseases, have increased in most countries. While many countries have or are planning to incorporate UPF recommendations in their national dietary guidelines, the classification of food processing levels relies on expertise-based manual categorization, which is labor-intensive and time-consuming. Our study utilized transformer-based language models to automate the classification of food processing levels according to the NOVA classification system in the Canada, Argentina, and US national food databases. We showed that fine-tuned language models using the ingredient list text found on food labels as inputs achieved a high overall accuracy (F1 score of 0.979) in predicting the food processing levels of Canadian food products, outperforming traditional machine learning models using structured nutrient data and bag-of-words. Most of the food categories reached a prediction accuracy of 0.98 using a fined-tuned language model, especially for predicting processed foods and ultra-processed foods. Our automation strategy was also effective and generalizable for classifying food products in the Argentina and US databases, providing a cost-effective approach for policymakers to monitor and regulate the UPFs in the global food supply.

## 1. Introduction

In recent decades, the availability of ultra-processed foods (UPFs) has increased in most countries, dominating the food supplies in high-income countries and rapidly increasing in middle-income countries [1,2]. Evidence from different countries has shown associations between a high consumption of UPFs with a poor diet quality, excess body weight, and other diet-related noncommunicable diseases (NCDs) [3,4,5,6,7]. Additionally, a high consumption of UPFs has been significantly associated with a higher risk of all-cause mortality among adults [8]. Recognizing this issue, many countries have incorporated recommendations in their national dietary guidelines to limit or avoid the consumption of UPFs. For instance, countries such as Belgium, Brazil, Chile, Ecuador, Israel, Malaysia, Maldives, Mexico, Peru, and Uruguay have included such recommendations [9,10,11,12]. Moreover, other countries, such as the US, are currently discussing UPFs as part of the development of the Dietary Guidelines for Americans 2025–2030 [13,14]. Therefore, in an ever-changing food supply, it is crucial to monitor its changes and understand the different impacts of a higher consumption of UPFs (nutritional, health, economic, and environmental, etc.). Thus, a timely assessment of this matter is key to having it on the public health agenda and contributing to evidence-based policy decision making. UPFs can be defined within the NOVA (not an acronym) food classification system. Under NOVA, foods are categorized into four groups according to the nature, extent, and purpose of their industrial processing (NOVA 1, unprocessed or minimally processed foods; NOVA 2, processed culinary ingredients; NOVA 3, processed foods; and NOVA 4, ultra-processed foods) [15]. UPFs are known for their hyper-palatability, affordability, convenience, long shelf life, and ready-to-consume nature. These products are characterized by a high energy density and low nutrient content. UPFs often contain added food additives that make the final product palatable or hyper-palatable, and are usually high in nutrients of public health concern (i.e., fats, sodium, and sugars) [1,15,16,17]. However, the categorization of foods under the NOVA system usually relies on manual categorization and matching [18], which is labor-intensive, time-consuming, and challenging given the dynamic food supply in most countries.

Machine learning (ML) is rapidly gaining popularity as a valuable tool among researchers in nutrition and public health policy. Previous studies have implemented machine learning and used inputs such as representing food ingredient appearance by binary numbers of 0 and 1 or the structured data of nutrient values for estimating label nutrients and food processing levels [19,20]. However, these extensive input requirements and computational resources often constrain the model performance, and these algorithms cannot easily process the other valuable unstructured text information found on food labels, such as name, brand, ingredients list, and nutrition claims. Of note, the textual information displayed on food packages is the easiest accessible information compared to complex nutrient composition data or laboratory food analytic reports, especially for many countries that lack a comprehensive nutrient composition database.

Recent advances in large language models (LLMs) for natural language processing (NLP) have provided a new possibility for extracting information from unstructured data. While evidence regarding the application of large language models in food- and nutrition-related tasks is relatively limited, these models have consistently demonstrated an expectational performance in tasks such as text classification, document summarization, question and answering, and generating interpretable explanations in a variety of domains [21,22,23]. Bidirectional Encoder Representations from Transformers (BERT) is one of the first developed transformer-based language models that is pre-trained on a large corpus of English data for downstream tasks such as categorization and similarity comparisons. Recent studies have indicated that pre-trained language models, such as BERT, have achieved excellent performances in food categorization and nutrition quality prediction by using the text information found on food labels [24,25]. Pre-trained representations have been shown to be generally transferable to various downstream tasks using a limited amount of nutrition label information. Thus, utilizing a pre-trained language model has the potential to fully exploit the unstructured text data found on food labels for NOVA food processing level classification and to reduce the number of inputs needed. In addition, pre-trained language models can be fine-tuned and applied to specific tasks with the benefit of learned features.

However, to the best of our knowledge, no study has applied a pre-trained language model and fine-tuned method to automate the food processing level classification in the global food supply. Therefore, this study aims to utilize a fine-tuning transformer-based language model to automate the classification of foods under the NOVA classification system for the foods available in the food supplies of Canada, Argentina, and the US.

## 2. Materials and Methods

### 2.1. Food Composition Databases

This study used the University of Toronto Food Label Information and Price Canada (FLIP-Canada) Database 2010–2020 (*n* = 118,985), the FLIP-Latin America and the Caribbean countries (FLIP-LAC) Database 2018–2022 (*n* = 8465), and the United States Department of Agriculture-Branded Food Products Database (USDA-BFPD) 2013–2022 (*n* = 1,702,235, *n* = 388,650 unique UPC) as inputs [18,26]. Briefly, FLIP is a database of Canadian and LAC branded packaged foods and beverages developed by the University of Toronto in 2010, which is updated every 3 to 4 years. It contains food label information (e.g., product name, brand, nutrition facts, ingredients, stores, price, and product images, etc.) for more than 120,000 food products from top food retailers in Canada and Latin America and the Caribbean countries. The FLIP dataset has been an essential research tool for monitoring changes in the food supply and informing the food policy-making process for more than 10 years. Previous versions of FLIP collected food labeling information manually or through a digital collection application (APP) [27]. The latest iteration for FLIP-Canada 2020 and FLIP-LAC 2022 (Argentina) collected food labeling information via website scraping and utilized optical character recognition (OCR) technology [18]. The USDA-BFPD is a publicly available US database that provides information on food and nutrient profiles for over 380,000 unique products. This information includes a product name and generic descriptor, serving size in g or mL, nutrients on the Nutrition Facts Panel per serving size and a 100 g/mL/oz basis, an ingredient list, and a date stamp associated with the product formulation.

### 2.2. NOVA Food Classification System

The processing levels of packaged foods and beverages are categorized under the NOVA food classification system, which includes four groups. NOVA 1: unprocessed or minimally processed foods; NOVA 2: processed culinary ingredients; NOVA 3: processed foods; and NOVA 4: ultra-processed foods [16]. The NOVA system of food classification is based on the nature, extent, and purpose of food processing to identify ultra-processed food products [15,28]. NOVA categories were manually assigned to foods in FLIP-Canada 2017 using methods that have been previously described [29]. Briefly, a trained nutrition researcher evaluated the list of ingredients for each food in FLIP-Canada 2017 and assigned them a NOVA food category, then a second researcher independently categorized a random 20% of the analytic sample. Weighted Cohen’s Kappa test was used to estimate the inter-rater reliability, which found an almost perfect agreement [29]. In addition, we manually determined the NOVA categories of randomly selected food products in the FLIP-LAC and USDA-BFPD databases to create validation subsets.

### 2.3. Data Preparation

**Figure 1:**
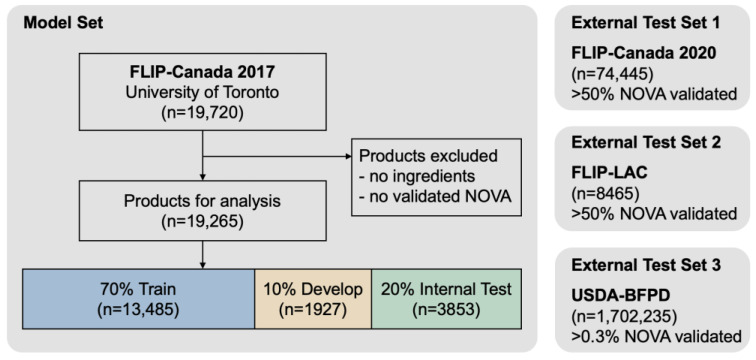
Data preparation flow chart and exclusion criteria *. * FLIP, University of Toronto Food Label Information and Price database. FLIP-Canada, FLIP-Canada database. FLIP-LAC, FLIP-Latin America and the Caribbean database. USDA-BFPD, United States Department of Agriculture-Branded Food Products Database.

Figure 1 describes the data preparation flow. A total of 19,720 products were extracted from FLIP-Canada 2017. For the NOVA food classification tasks, we excluded products that did not contain ingredient information and did not have a validated NOVA category. The final sample size for the FLIP-Canada 2017 NOVA classification tasks was 18,916, and all the products were manually validated for NOVA by a trained nutrition researcher. In addition, we used FLIP-Canada 2020 as a validation dataset (*n* = 74,445, >50% manually validated for NOVA by a trained nutrition researcher) to validate the machine learning algorithms developed from FLIP-Canada 2017. Furthermore, we applied the algorithm to the prediction datasets FLIP-LAC 2018–2022 for Argentina (*n* = 8465, >50% manually validated for NOVA by a trained nutrition researcher) and USDA-BFPD (*n* = 1,702,235, >0.3% manually validated for NOVA by a trained nutrition researcher).

### 2.4. Food Representations

A pre-trained language model (i.e., sentence-BERT) was used to convert the ingredient text lists on food labels into low-dimensional dense vector representations. In addition, a bag-of-words (BoW) representation (i.e., the presence of each ingredient in the given ingredients list) and structured nutrient fact data (i.e., the amount of nutrients per 100 units in the nutrition facts table) were used as inputs [24]. All the text in the ingredients list was cleaned and converted into capitals, separated by commas. The nutrient values displayed in the Nutrient Fact table (NFt) were standardized into 100 units (g for solid food products and mL for liquid food products). To utilize the extracted representations for the NOVA food classification, we used extreme gradient boosting (XGBoost) algorithms.

### 2.5. Fine-Tuning Language Model

We utilized pre-trained language models and added a classification layer on top. The entire model was then fine-tuned end-to-end using specified datasets. We used the BERT-Base, DistilBERT-Base, MPNet-Base, MiniLM-L6, and multi-qa-MiniLM-L6-cos classification algorithms [30]. These pre-trained models vary in size, training data source, and number of encoder layers stacked on top of each other, which, in turn, affects their run-time and prediction quality. Fine-tuned models, in comparison to linear probing, have the advantage of being able to adapt pre-trained representations to a given dataset. This adaptability often results in an improved performance, but it also requires more computation resources to train an entire model.

### 2.6. Statistical Analyses

The performance of the model on the given NOVA classification tasks was measured using different parameters, including accuracy, balanced accuracy, F1 score, confusion matrix, normalized confusion matrix, receiver operating characteristic (ROC) curve, and area under the curve. Accuracy was the ratio of correctly predicted observations to the total observations, which mainly depended on the performance that the algorithm achieved on the biggest classes. Balanced accuracy is useful for multi-class classification when classes are imbalanced, and each class will have an equal weight in the final calculation [31]. F1 score was the weighted average of precision (positive values which were gained from the prediction, relevant occurrences among the gained occurrences) and recalls (relevance of gained occurrences). The confusion matrix contained true positive, true negative, false positive, and false negative values in the matrix, which were used to evaluate the actual values with the values predicted by the classifier. Confusion matrix normalization by the number of elements in each class displayed a more visual interpretation in the case of class imbalance. ROC is a probability curve plot and a higher area under the curve represented a higher ability of the model to distinguish between classes. All the analyses were conducted using Python 3.9.

## 3. Results

### 3.1. Different Machine Learning Algorithms Reached Moderate to High Accuracy in NOVA Food Processing Levels Classification

Table 1 shows the results of the NOVA classification algorithms using different food label representations and probing methods. The performance of each classifier was measured in terms of its accuracy, balanced accuracy, and F1 score. Using the nutrition levels indicated in the Nutrition Facts table (NFt) to predict the NOVA classification reached a moderate accuracy (accuracy 0.890, balanced accuracy 0.797, and F1 score 0.882). Using the ingredient list information found on the food labels (bag-of-words and pre-trained embeddings methods) predicted a more accurate NOVA food classification than using the nutrient information in the nutrition fact table (structured nutrient facts model). By using the XGBoost classifier, the highest performances of the bag-of-words (accuracy 0.970, balanced accuracy 0.938, and F1 score 0.970) and pre-trained embeddings (accuracy 0.940, balanced accuracy 0.882, and F1 score 0.940) methods were better than that of the structured nutrient facts model method.

### 3.2. Fine-Tuned Language Model Performed Well in NOVA Food Processing Levels Classification

**Figure 2:**
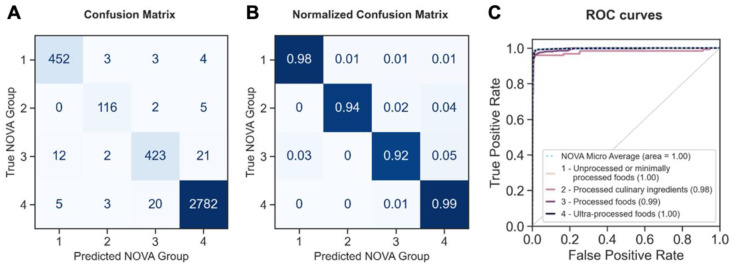
The performance of NOVA classification using a fine-tuned language model *. (**A**) Confusion matrix. (**B**) Normalized confusion matrix. (**C**) Receiver operating characteristic (ROC) curves and aera under the ROC. * NOVA Group 1, unprocessed or minimally processed foods; NOVA Group 2, processed culinary ingredients; NOVA Group 3, processed foods; and NOVA Group 4, ultra-processed foods.

The fine-tuned language model performed the best among the different machine learning algorithms (Table 1), with an excellent accuracy of 0.978–0.979, balanced accuracy of 0.955–0.959, and F1 scores of 0.978–0.979. Within the fine-tuned language model, the multi-qa-MiniLM-L6-cos-v1 model (with a 512 max sequence length, 384 dimensions, and approximately 80 MB size) had the highest accuracy, balanced accuracy, and F1 score compared to other pre-trained, fine-tuned models. BERT-Base had an accuracy, balanced accuracy, and F1 score of 0.978, 0.955, and 0.978, respectively. The accuracy, balanced accuracy, and F1 score of the DistilBERT-Base model were 0.978, 0.955, and 0.978, respectively. The MPNet-Base model and all-MiniLM-L6 model had the same accuracy and F1 score as DistilBERT-Base, but a lower balanced accuracy (0.954 and 0.956, respectively). Figure 2 shows the confusion matrix, normalized confusion matrix, and ROC curves of each NOVA food category using the multi-qa-MiniLM-L6-cos-v1 model. Specifically, the fine-tuned language model reached an accuracy of 0.98 for unprocessed or minimally processed foods, 0.94 for processed culinary ingredients, 0.92 for processed foods, and 0.99 for ultra-processed foods. The fine-tuned language model showed a high area under the curve (0.98–1.00) in ROC, indicating robust classification capabilities.

**Figure 3:**
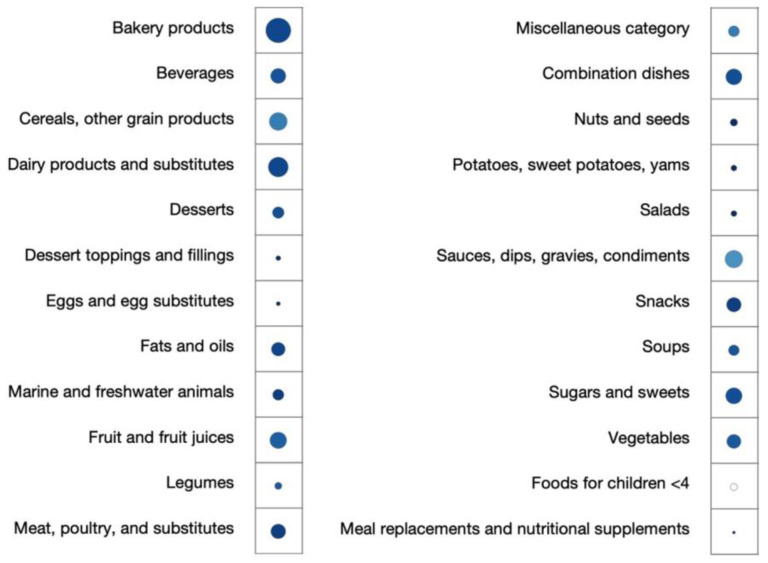
Accuracy of NOVA classification prediction by food categories using a fine-tuned language model *. * Bubble size depicts the relative sample size of each food category predicted by the fine-tuned language model in internal test dataset. Bubble color density indicates the overall accuracies in terms of F1 scores.

Different food categories have shown an overall NOVA prediction accuracy range from 0.954 to 1, except for foods for children under four years of age (0.889) (Figure 3). Salads, eggs, nuts, potatoes, dessert toppings and fillings, meal replacements, and nutritional supplements reached the overall accuracy of 1. The overall accuracy of major food categories such as meat, snacks, dairy, bakery, and beverages was higher than 0.98. Among the four NOVA groups, 9 out of 24 food categories in NOVA Group 1 (unprocessed or minimally processed foods) and 7 food categories in NOVA Group 3 and NOVA Group 4 (processed foods and ultra-processed foods) reached an F1 score of 1. In addition, 80% of the food categories in of ultra-processed foods reached an F1 score of 0.98, indicating a high prediction performance using the fine-tuned language model.

### 3.3. The Generalization Ability of the Fine-Tuned Language Model in NOVA Food Processing Levels Classification

We applied the fine-tuned language model (developed based on a fully validated FLIP-Canada 2017 database) to the FLIP-Canada 2020, FLIP-LAC (Argentina), and USDA-BFPD datasets. The results indicated that the fine-tuned language model maintained a high accuracy depending on the source of data, which outperformed the structured nutrient facts, bag-of-words, and pre-trained language models (Table 2). Using subset of >50% food products with manually validated NOVA categories in FLIP-Canada 2020, the fine-tuned language model reached a 0.941 accuracy, 0.896 balanced accuracy, and 0.940 F1 score. The fine-tuned language model also performed moderately well when applied to randomly selected >50% FLIP-LAC and >0.3% USDA-BFPD database subsets (with manually validated NOVA categories), and reached F1 scores of 0.734 for FLIP-LAC and 0.947 for USDA-BFPD, respectively. Of note, when we trained the fine-tuned model on the FLIP-LAC 2022 database, it reached an F1 score of 0.889 (Table 2).

### 3.4. Using Fine-Tuned Language Model to Estimate NOVA Food Processing Levels in the Food Supply across Different Countries

**Figure 4:**
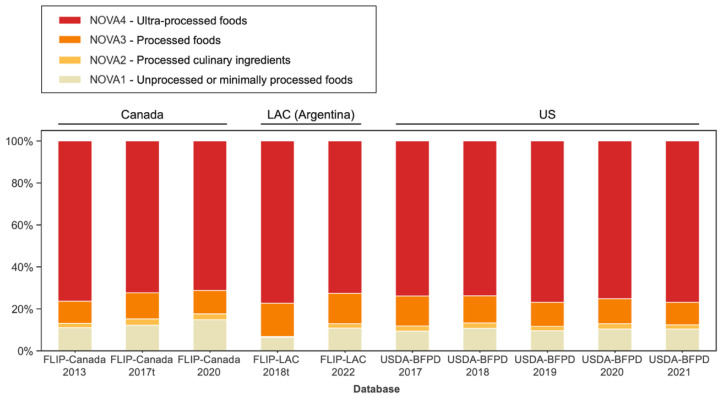
Fine-tuned model prediction of NOVA category using food label text embeddings in FLIP-Canada, FLIP-LAC, and USDA-BFPD food composition databases *^,†^. * FLIP, University of Toronto Food Label Information and Price database. FLIP-LAC, FLIP-Latin America and the Caribbean database. USDA-BFPD, United States Department of Agriculture-Branded Food Products Database. ^†^ FLIP-Canada 2017t reports the true NOVA proportions by manually assigned and validated categories. FLIP-LAC 2018t reports the true NOVA proportions by manually assigned and validated NOVA categories.

The fine-tuned language model predicted a prevalence of ultra-processed foods (NOVA 4) between 76.3% (FLIP-Canada 2013), 72.3% (FLIP-Canada 2017t), and 71.3% (FLIP-Canada 2020) in the FLIP Canadian databases (Figure 4). There were 77.4% (FLIP-LAC 2018t) and 72.6% (FLIP-LAC 2022) ultra-processed foods determined in the FLIP-LAC Argentina databases (Figure 4). The prevalence of ultra-processed foods was 73.9% (USDA-BFPD 2017), 73.8% (USDA-BFPD 2018), 76.9% (USDA-BFPD 2019), 75.1% (USDA-BFPD 2020), and 76.9% (USDA-BFPD 2021) in the examined US branded food databases (Figure 4).

Moreover, between 11.2%, 2.7%, 14.9% (FLIP-Canada 2020), 14.4%, 2.1%, and 10.8% (FLIP-LAC 2022) and 10.7%, 2.0%, and 10.4% (USDA-BFPD 2021) of foods were classified as processed foods (NOVA 3), processed culinary ingredients (NOVA 2), and unprocessed or minimally processed foods (NOVA 1), respectively (Figure 4).

## 4. Discussion

The findings from this study first demonstrated that the fine-tuned language model using ingredient lists as inputs performed well in predicting NOVA food processing categories. Our results showed that the fine-tuned language model reached a 0.979 F1 score for NOVA food classification, and this model is generalizable and presents a moderately high accuracy (F1 scores of 0.889–0.947) when applied to different food composition datasets from Canada, the US, and Argentina.

Our model predicted that the Canadian food supply primarily comprises UPFs (71.3% in the FLIP-Canada 2020 database). Previous work by our research group manually classified packaged food and beverage products in FLIP-Canada 2017 under the NOVA classification system, and 73.5% were classified as UPFs [29]. It is worth noting that FLIP excludes products that are not required to display a Canadian nutrition fact table (i.e., fresh fruits and vegetables, raw meats, and seafood). Therefore, the prevalence of these foods in the Canadian food supply, usually classified under NOVA 1 and NOVA 2, were most likely underestimated [29]. Furthermore, the NOVA classification system itself has faced scientific debate, and previous research has shown that it is more consistent for certain foods than for others [32,33]. Studies assessing the healthiness of packaged food and beverage products in the US found that between 71% and 73% of them were UPFs [20,34], similar to our estimated results (between 73.8% and 76.9%), although different US food databases were used in these studies.

The dietary and health outcomes due to a high consumption of UPFs, as defined by the NOVA system, have been widely studied [35]. Emerging evidence shows that a high consumption of UPFs has been associated with a worse cardiometabolic risk profile and higher risk for cardiovascular diseases, depression, and all-cause mortality [35]. In Canada, UPFs contribute to more than 45% of the total daily energy intake on average [36], and most of the calories derived from free-sugar intake (71.5%) come from UPF consumption [37]. In the US, the contribution of UPFs to the total daily energy intake went from 53.5 to 57.0% kcal between 2001 and 2018 [38]. In Argentina, UPFs contribute to more than 25% of the total daily energy intake [39]. The availability and consumption of UPFs dominate the food supplies in high-income countries, such as Canada and the US, and are rapidly increasing in middle-income countries such as Argentina [1,2]. Therefore, efficiently monitoring the availability of UPFs in the food supply and identifying the most problematic food categories are key to informing future policy decisions aimed at improving food environments, diet quality, and protecting the population from the harmful health effects of a high consumption of UPFs.

Classifying foods under the NOVA classification system primarily relies on manual categorization and validation by trained nutrition researchers. This process is labor-intensive and time-consuming. For instance, traditional approaches to identifying UPFs involve assessing the ingredients list, especially looking for food substances rarely used in kitchens (i.e., hydrolyzed proteins, soya protein isolate, gluten, casein, whey protein, mechanically separated meat, fructose, high-fructose corn syrup, fruit juice concentrate, invert sugar, maltodextrin, and dextrose, etc.) or other food additives that make the final product palatable or hyper-palatable (i.e., flavors, flavors enhancers, colors, emulsifiers, emulsifying salts, sweeteners, and thickeners, etc.), which are usually present in UPFs [15].

Machine learning provides a powerful tool for food classification and nutrition quality prediction tasks. An earlier study indicated that a pre-trained language model and supervised machine learning accurately predicted packaged food category and nutrition quality using the text information found on food labels [24]. Since the NOVA food classification system is mainly based on the appearance of target ingredients, our results showed that the bag-of-words model performed slightly better than the pre-trained language model and outperformed the structured nutrient facts model. A recent study utilized nutrition concentrations as inputs and applied a machine learning algorithm based on a multi-class random forest classifier to accurately predict the degree of food processing in a US food composition database (i.e., The Food and Nutrient Database for Dietary Studies, USDA-FNDDS) [20]. This method was developed based on a sample size of 2484 food items and relied on complex nutrient information (an input of 99 nutrients, e.g., retinol, riboflavin, and total polyunsaturated fatty acids, etc.). This automation algorithm reached a high accuracy and offers a great solution for predicting the food processing levels for products without ingredient lists. However, for many other countries, comprehensive food composition data are hard to collect and standardize, most of which are rarely identified by consumers, whereas ingredient text lists are available and quite standardized in nearly all packaged food products worldwide.

Compared to existing methods, the strength of our strategy is that it provides a direct and powerful machine learning tool to utilize the text information available on the food labels displayed on food packages (i.e., the ingredient list and nutrition facts tables) for the prediction of NOVA food processing levels. Our model was trained on about 20,000 food products that were manually assessed and validated under the NOVA food classification system. This algorithm directly utilized the single text of ingredient lists for the fine-tuned language model and achieved a higher accuracy, which made more products available for training, including food products with missing nutritional information. When the ingredients list was missing, using the remaining nutrient information displayed in the nutrition facts table (e.g., calories, fat, sodium, fiber, sugars, and protein, etc., 14 in total) and the traditional machine learning algorithm still provided a moderate accuracy. Thus, our algorithm provided a fast and accurate assessment of the NOVA food processing classification, which is necessary for monitoring the dynamic and ever-changing food supply and could facilitate cross-country comparisons, where extensive nutrition information may be lacking. Coupled with recent e-grocery trends and methodologies used to collect food label information through web-scrapping and OCR, our strategy largely reduced the manual work of food processing level classification under NOVA and other systems. In addition, our algorithm is generalizable and can be applied to food composition databases with varied information collected from different countries, especially countries without extensive food composition information in their food database.

However, our method is not perfect and its performance on new datasets could decrease, which indicates that further training on new datasets is necessary and some manual validation on the appropriate proportion of predicted data is still needed. The performance of our model was also limited by different local languages and the accuracy of the optical character recognition text recognition. For example, for the FLIP-LAC Argentina database, the algorithm was based on translated ingredient lists from Spanish to English; therefore, the model accuracy slightly decreased when applying our algorithm to other countries. Future research could explore the incorporation of stemming techniques and the development of food-specific corpora to improve the recognition of food ingredients and enhance the overall model performance across diverse linguistic and cultural contexts. Another limitation of this study is that our data sample in FLIP and USDA-BFPD databases did not cover 100% of the food products available in the food supply. Although the databases used in this analysis still have a very good coverage rate of the food supply (e.g., 80% in FLIP), future work is needed to improve the challenge of the timely collection of food label information in a country.

This study demonstrated that using large language models is an effective and generalizable automation strategy for classifying the NOVA food processing levels of packaged foods. Our automation strategy can be applied to different countries to expedite the food categorization process under the NOVA classification system globally, given that the ingredient text lists on food packages are the easiest accessible data compared to more complex nutrient composition data. Our approach could have profound policy implications. By facilitating a faster and more efficient NOVA ultra-processed food categorization process, we can assist researchers and policymakers in monitoring the changes of UPFs in food supply. Moreover, it could enhance our understanding of the correlations between NOVA food processing levels and health outcomes, which can inform future policy decisions aiming to improve food environments, diet quality, and public health on a global scale.

## Figures and Tables

**Table 1:** Accuracy, balanced accuracy, and F1 score of NOVA classification algorithms using different methods.

Feature *	Model ^†^	NOVA Classification Performance		
		Accuracy	Balanced Accuracy	F1 Score
Structured data	XGBoost	0.890	0.797	0.882
Bag-of-words	XGBoost	0.970	0.938	0.970
Pre-trained embeddings	XGBoost	0.940	0.882	0.940
Fine-tuned language models	BERT-Base	0.978	0.955	0.978
	DistilBERT-Base	0.979	0.958	0.979
	MPNet-Base	0.979	0.954	0.979
	all-MiniLM-L6	0.979	0.956	0.979
	multi-qa-MiniLM-L6-cos	0.979	0.959	0.979

* Structured data, nutrition levels per 100 units as input. Bag-of-words, top 2000 ingredients as input. Pre-trained embeddings, modified pre-trained BERT model using ingredient list as input. Fine-tuned language models, ingredient list as input. Models were trained on FLIP-Canada 2017 dataset. ^†^ XGBoost, extreme gradient boosting. Fine-tuned language models (bert-base, distilbert-base, all-mpnet-base-v2, all-MiniLM-L6-v2, and multi-qa-MiniLM-L6-cos-v1), epoch = 10.

**Table 2:** Generalization performance of food NOVA category prediction models using validated subset of FLIP-Canada, FLIP-LAC, and USDA-BFPD databases.

Method *	Database ^†^	NOVA Classification Performance		
		Accuracy	Balanced Accuracy	F1 Score
Structured data	FLIP-Canada	0.872	0.737	0.862
Bag-of-words	FLIP-Canada	0.937	0.870	0.936
Pre-trained embedding	FLIP-Canada	0.921	0.831	0.917
Fine-tuned model	FLIP-Canada	0.941	0.896	0.940
Structured data	FLIP-LAC	0.857	0.726	0.850
Bag-of-words	FLIP-LAC	0.863	0.681	0.858
Pre-trained embedding	FLIP-LAC	0.825	0.567	0.808
Fine-tuned model	FLIP-LAC	0.891	0.654	0.889
Structured data	USDA-BFPD	0.806	0.647	0.788
Bag-of-words	USDA-BFPD	0.919	0.832	0.918
Pre-trained embedding	USDA-BFPD	0.900	0.766	0.892
Fine-tuned model	USDA-BFPD	0.948	0.881	0.947

* Structured data, nutrition levels per 100 units. Bag-of-words, top 2000 ingredients. Pre-trained embeddings, modified pre-trained BERT model using ingredients. All used the XGBoost classifier. Fine-tuned model used multi-qa-MiniLM-L6-cos-v1, epoch = 10. ^†^ FLIP, University of Toronto Food Label Information and Price database. FLIP-Canada 2020 validated subset was used. FLIP-LAC, FLIP-Latin America and the Caribbean database, FLIP-LAC 2022 validated subset was trained. USDA-BFPD, United States Department of Agriculture-Branded Food Products Database, validated subset was used.

## Data Availability

All relevant data supporting the findings of this study are available within the paper. Source databases will be available upon request pending application and approval.
